# Multimatrix surveillance of multidrug-resistant and ESBL-producing *Klebsiella pneumoniae* in dairy farm ecosystems: A One Health study in Batu City, Indonesia

**DOI:** 10.14202/vetworld.2026.1342-1355

**Published:** 2026-03-28

**Authors:** Fidi Nur Aini Eka Puji Dameanti, Sheila Marty Yanestria, Emmanuel Nnabuike Ugbo, Aswin Rafif Khairullah, Mustofa Helmi Effendi, Rahayu Sutrisno, Muhammad Ali Akramsyah Safri, Indah Amalia Amri, Siti Kurniawati, Sruti Listra Adrenalin, Tira Erlinda, Na Young Nirmalasari, Bagus Aji Masardhi, Nadia Ananda Prasetia Dion

**Affiliations:** 1 Laboratory of Veterinary Microbiology and Immunology, Faculty of Veterinary Medicine, Universitas Brawijaya, Malang 65151, East Java, Indonesia; 2 Department of Veterinary Public Health, Wijaya Kusuma Surabaya University, Surabaya 60225, East Java, Indonesia; 3 Department of Applied Microbiology, Faculty of Science, Ebonyi State University, Abakaliki 480211, Nigeria; 4 Research Center for Veterinary Science, National Research and Innovation Agency (BRIN), Jl. Raya Bogor Km. 46, Cibinong, Bogor 16911, West Java, Indonesia; 5 Department of Veterinary Public Health, Faculty of Veterinary Medicine, Universitas Airlangga, Surabaya 60115, East Java, Indonesia; 6 Research Group of Antimicrobial Resistance in Faculty of Veterinary Medicine, Universitas Airlangga Surabaya, East Java, Indonesia; 7 School of Food Industry, Faculty of Bioresources, and Food Industry, Universiti Sultan Zainal Abidin (Besut Campus), Besut, 22200; 8 Master of Nutrition Science Study Program, Faculty of Health Sciences, Universitas Brawijaya, Jl. Puncak Dieng, Kalisongo, Malang Regency 65151, East Java, Indonesia; 9 Student on Faculty of Veterinary Medicine, Universitas Brawijaya, Jl. Puncak Dieng, Kalisongo, Malang Regency 65151, East Java, Indonesia

**Keywords:** antimicrobial resistance, dairy farm, extended-spectrum beta-lactamase, Indonesia, *Klebsiella pneumoniae*, multidrug resistance, One Health, SDG, surveillance

## Abstract

**Background and Aim:**

Antimicrobial resistance (AMR) in extended-spectrum β-lactamase (ESBL)–producing *Klebsiella pneumoniae* is a growing global public health concern, especially at the human–animal–environment interface of dairy farming systems. Dairy farms may serve as reservoirs for resistant bacteria through contaminated milk, feed, water, soil, and human-related environmental sources, enabling transmission within a One Health framework. This study aimed to determine the prevalence of AMR, multidrug resistance (MDR), and ESBL-producing *K. pneumoniae* across various matrixes in dairy cattle farms in Batu City, East Java, Indonesia, to identify potential on-farm reservoirs and transmission pathways.

**Materials and Methods:**

A cross-sectional surveillance study was conducted from May to August 2025 on 59 dairy farms. One sample per matrix per farm was collected, including milk, forage, soil, animal drinking water, hand-wash water, and feces (total n = 354). Isolation and phenotypic identification of *K. pneumoniae* were carried out using standard microbiological and biochemical methods. Antimicrobial susceptibility testing against seven antibiotics representing different classes was performed using the Kirby–Bauer disk diffusion method following Clinical and Laboratory Standards Institute guidelines. Isolates resistant to ≥3 antimicrobial classes were classified as MDR, and ESBL production was confirmed using the double-disk synergy test. Prevalence estimates were calculated with 95% confidence intervals.

**Results:**

*K. pneumoniae* was found in 61.3% (217/354) of all samples, with the highest occurrence in forage feed (84.7%), drinking water (74.6%), hand-wash water (71.2%), and soil (71.2%), followed by milk (50.8%) and feces (15.3%). High resistance rates were seen for ampicillin (89.4%), streptomycin (71.4%), and cefotaxime (38.3%), while resistance to ciprofloxacin was low (2.8%). MDR was present in 35.0% (76/217) of isolates, most commonly showing resistance to three antimicrobial classes (61.8%). Among the MDR isolates, 21.1% (16/76) were confirmed as ESBL producers, mainly from environmental sources, especially forage feed and drinking water.

**Conclusion:**

The widespread detection of MDR and ESBL-producing *K. pneumoniae* in animal-derived, environmental, and human-related samples shows that dairy farms serve as significant One Health reservoirs of antimicrobial resistance. Environmental factors, particularly feed and water, seem to play a crucial role in the persistence and spread of resistance. These results provide baseline epidemiological data for Indonesian dairy farms and emphasize the importance of better antimicrobial stewardship, improved farm biosecurity, and future molecular surveillance to better understand resistance patterns and support risk-based strategies to reduce AMR dissemination in dairy production ecosystems.

## INTRODUCTION

Dairy cattle farming is vital to Indonesia’s food system, making the health of animals and milk quality crucial for producing safe, high-quality food for people [1, 2]. East Java is among the top provinces in the country for dairy cattle numbers and milk output, with Batu City contributing significantly by producing about 25,258 liters of milk daily [2, 3]. Many dairy farms in Batu City also serve as educational agrotourism spots, fostering frequent interactions among livestock, farm settings, and the public. These public-facing farms may increase exposure to antimicrobial-resistant (AMR) bacteria, positioning dairy farms as key points in the spread of antimicrobial resistance within a One Health framework. The detection of multidrug-resistant (MDR) and extended-spectrum β-lactamase (ESBL)-producing *Klebsiella pneumoniae* in dairy farm environments poses a potential One Health risk that could affect not only farm workers but also consumers and visitors.

The quality of milk depends heavily on proper herd health management and disease control practices, which often involve the use of antibiotics for treatment and prevention [4, 5]. However, the widespread and uncontrolled use of antibiotics in dairy farming has led to the global rise of antimicrobial resistance, reducing treatment effectiveness, extending disease duration, and increasing the spread of antibiotic residues and resistance genes in the environment [6–8]. According to the Global Research on Antimicrobial Resistance report, infections caused by resistant bacteria resulted in about 4.95 million deaths worldwide in 2019, with forecasts pointing to significant impacts on global health and livestock production by 2050 [9, 10]. A key mechanism behind antimicrobial resistance is the production of enzymes that break down β-lactam antibiotics, making bacteria resistant to third-generation cephalosporins and similar drugs [11, 12]. ESBL production is often linked with MDR, as ESBL-producing bacteria frequently show resistance to other antibiotic classes, including aminoglycosides and chloramphenicol [13, 14]. Among Enterobacteriaceae, *K. pneumoniae* is a major ESBL producer and a common organism found in humans, animals, and environmental sources [10, 15]. Studies have found ESBL-producing bacteria in milk, soil, water, feces, wastewater, and manure [16–19]. These sources support the spread of antimicrobial resistance genes, which are often carried on mobile genetic elements and can transfer horizontally among bacteria in animals, the environment, and humans [19–22]. In Indonesia, where dairy farming is mainly small-scale and antibiotics are often used empirically, penicillins, tetracyclines, and fluoroquinolones are among the most common antibiotics used in food-producing animals, raising the likelihood of resistance development and environmental contamination [23–28]. Widiastuti *et al*. [25] noted that tetracyclines were the most used antibiotics on dairy farms in Boyolali (Central Java) and Malang (East Java), with doxycycline residues found in milk samples. Additionally, a wastewater study in Batu City revealed high resistance levels to chloramphenicol, cefotaxime, ampicillin, and streptomycin [28]. Despite these findings from clinical and wastewater surveillance, the environmental reservoirs within dairy production systems at the farm-level are still poorly understood.

Despite rising reports of antimicrobial resistance in livestock systems, most studies in Indonesia have concentrated on clinical isolates, milk samples, or wastewater surveillance, with limited focus on the broader farm ecosystem where resistant bacteria may persist and circulate. Dairy farms are complex environments where animals, feed, water, soil, equipment, and human-contact surfaces continually interact, creating multiple opportunities for resistant bacteria to be maintained and spread. However, data on the occurrence of multidrug-resistant and extended-spectrum β-lactamase-producing *K. pneumoniae* across various farm matrices remain limited, especially at the farm-level before environmental dilution takes place. Previous research has seldom used a multimatrix surveillance approach that evaluates animal, environmental, and human-contact interfaces within the same farm system. Additionally, forage feed and water sources, which may serve as significant but underrecognized reservoirs of resistant bacteria, have not been sufficiently studied in Indonesian dairy farm antimicrobial resistance research. The lack of such integrated data restricts understanding of transmission pathways and hampers the creation of targeted intervention strategies within a One Health framework.

Therefore, the present study was carried out to conduct a comprehensive multimatrix surveillance of antimicrobial resistance, multidrug resistance, and extended-spectrum β-lactamase-producing *K. pneumoniae* in dairy cattle farm ecosystems in Batu City, East Java, Indonesia. This study analyzed milk, forage feed, soil, animal drinking water, hand-wash water, and fecal samples to identify potential on-farm reservoirs and transmission interfaces of resistant bacteria. Using a One Health surveillance approach, the study aimed to establish baseline epidemiological data on the distribution of resistant *K. pneumoniae* within dairy production systems, assess the role of environmental matrixes in resistance persistence, and identify critical control points to help develop antimicrobial stewardship programs, risk-based biosecurity measures, and future molecular surveillance strategies for reducing antimicrobial resistance in dairy farm environments.

## MATERIALS AND METHODS

### Ethical approval

Ethical approval for this study was obtained from the Health Research Ethics Committee (Komite Etik Penelitian Kesehatan, KEPK), Universitas Muhammadiyah Malang, Indonesia, under approval number E.5.a/065/KEPKUMM/IV/2025. Before sample collection, permission to conduct the study was obtained from the relevant local authority in Batu City and from the owners or managers of all participating dairy farms. Written or verbal informed consent was obtained from farm owners or responsible personnel before enrollment in the study.

All sampling procedures were performed using non-invasive methods and in accordance with accepted animal welfare principles. Milk samples were collected during routine milking procedures after standard udder hygiene practices, whereas fecal samples were collected as freshly voided material from the ground without restraining or disturbing the animals. Environmental samples, including forage feed, soil, animal drinking water, and hand-wash water, were collected without causing harm, stress, or disruption to the animals or farm activities. No experimental infection, invasive handling, or therapeutic intervention was performed on the animals as part of this study.

All field and laboratory procedures were carried out in accordance with biosafety and biosecurity measures to minimize contamination risks and protect animals, farm workers, and researchers. The confidentiality of farm identity and participant information was maintained throughout the study, and the collected data were used solely for research purposes.

### Study design, period, and location

This study used a cross-sectional, surveillance-based approach to examine the presence of MDR and ESBL-producing *K. pneumoniae* in dairy farm environments. It was carried out between May and August 2025 on select small- to medium-scale dairy farms in Batu City, East Java Province, Indonesia. Batu City is a key dairy-producing region characterized by a high density of smallholder farms, some of which serve as educational agrotourism sites, leading to frequent interactions among livestock, farm workers, visitors, and the surrounding environment.

The study focused on farm-level environmental surveillance rather than investigating clinical cases, with sampling designed to capture multiple on-farm matrixes representing animal-derived products, environmental reservoirs, and human-associated interfaces. This approach was adopted to support a One Health surveillance framework by assessing potential AMR reservoirs and transmission pathways within dairy production systems before environmental dissemination occurs downstream.

### Farm selection, eligibility criteria, and sample size

During the study period, dairy farms were chosen based on convenience and voluntary participation. Farms in Batu City were identified through local dairy farmer associations and cooperative networks and invited to join the study. A total of 59 dairy farms agreed to participate and were included.

Farms were eligible for inclusion if they: (i) were actively producing raw cow’s milk during the study period; (ii) operated as small- to medium-sized dairy farms; (iii) allowed access for milk and environmental sample collection; and (iv) obtained informed consent from farm owners or responsible workers. Farms were excluded if they were not in active production, declined participation, or did not permit comprehensive sampling across the targeted matrixes.

A single-proportion formula was used to estimate the sample size for cross-sectional prevalence studies. Assuming an expected ESBL prevalence of 11% [29], a 95% confidence level (Z = 1.96), and a precision of 10%, the minimum required sample size was calculated to be 38 farms. The inclusion of 59 dairy farms exceeded this minimum requirement and was considered sufficient to generate baseline surveillance data for *K. pneumoniae*-producing MDR and ESBL. However, since farm participation was voluntary and non-random, the findings may not be fully representative of all dairy farms in the region, which is acknowledged as a study limitation.

### Sample collection, transport, and storage

To reduce contamination and ensure reproducibility across farms, sample collection was performed using standardized protocols. Samples were collected once per farm during the study period, with one sample taken for each predefined matrix per farm to broadly represent potential AMR within each dairy farm ecosystem. This method was chosen to support baseline surveillance at the farm-level rather than within-farm quantitative comparisons. To prevent cross-contamination, sterile tools were used for each sample, disposable gloves were changed between different sample types, and all samples were handled aseptically during collection and transport.

Milk samples (30 mL) were collected from individual lactating cows during routine milking after discarding the initial streams of milk and following standard udder hygiene procedures. The samples were stored in sterile containers. Animal drinking water samples (1,000 mL) were collected directly from water troughs used by the cattle, while hand-washing water samples (200 mL) were taken from designated hand-washing stations after milking to represent human-associated environmental exposure on the farm.

Forage feed samples (200 g) were aseptically collected from feed bunks or feed storage areas commonly accessed by cattle. Soil samples (25 g) were taken from areas around cattle housing at an approximate depth of 10 cm and within 1–2 m of the barn to represent the immediate farm environment. Freshly voided fecal samples (10 g) were gathered from the ground to minimize environmental contamination.

Six sample types were collected from each farm, resulting in 354 samples from 59 participating farms. All samples were immediately placed in sterile containers, stored in insulated cool boxes with ice packs, and transported under cold-chain conditions. The samples’ temperatures were monitored using calibrated digital thermometers inside the cool boxes, with readings recorded at the start of transport and upon arrival at the lab to ensure they stayed at approximately 4°C throughout transportation [30]. All samples were delivered to the Veterinary Microbiology and Immunology Laboratory, Faculty of Veterinary Medicine, Universitas Brawijaya, and processed within 6–12 h of collection. Sampling was conducted after obtaining permission from the relevant farm owners and local authorities.

### Isolation and phenotypic identification of *K. pneumoniae* strains

All collected samples were inoculated into Buffered Peptone Water (BPW) (Oxoid, Basingstoke, UK) at a 1:9 ratio and then incubated at 37°C for 24 h. Samples were pre-enriched in BPW with a 1:9 sample-to-broth ratio to improve the recovery of stressed or low-abundance bacteria and to neutralize any inhibitory substances in environmental and milk matrixes. BPW was chosen as a non-selective pre-enrichment medium according to ISO (International Organization for Standardization) and FDA BAM (Food and Drug Administration Bacteriological Analytical Manual) protocols [31].

The BPW isolates were then cultured on MacConkey Agar (MCA) (Oxoid). Colonies displaying mucoid morphology, presumed to be *K. pneumoniae*, were subsequently subcultured onto Eosin Methylene Blue Agar (EMBA) (Oxoid) and incubated at 37°C for 24 h [32]. Colonies with a pink, mucoid appearance were further identified through Gram staining and biochemical tests, including IMViC (Indole, Methyl Red, Voges–Proskauer, Citrate), Triple Sugar Iron Agar (TSIA), Sulfide Indole Motility (SIM), and urease. All media used for biochemical testing were sourced from Himedia, India [33]. The Gram staining procedure was performed according to Tripathi *et al*. [33].

**Figure 1:**
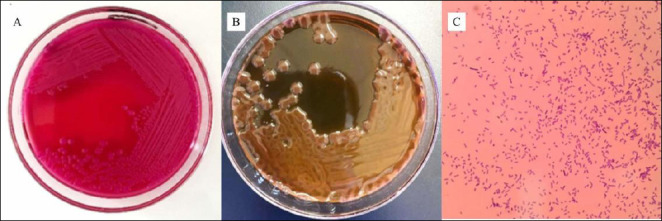
Isolation and identification of *Klebsiella pneumoniae*. (A) MacConkey Agar, (B) Eosin Methylene Blue Agar, and (C) Gram staining at 1000× magnification. Note: Yellow boxes indicate colonies and bacteria suspected to be *K. pneumoniae*.

**Figure 2:**
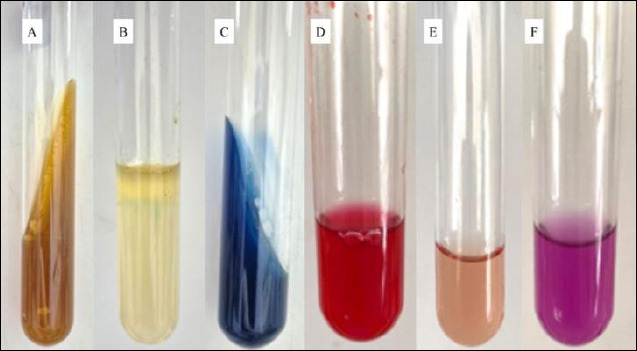
Identification results of *Klebsiella pneumoniae* in Triple Sugar Iron Agar, Indole–Methyl Red–Voges–Proskauer–Citrate biochemical tests, and urease test. (A)Triple Sugar Iron Agar, (B) Sulfide indole motility medium, (C) Simmons citrate agar, (D) Methyl red test, (E) Voges–Proskauer test, and (F) Urease test.

*K. pneumoniae* colonies on MCA appeared pale red to pink, with smooth edges, a convex surface, and a mucoid texture due to the presence of a thick polysaccharide capsule (Figure-1a). Lactose fermentation with subsequent acid production produced pink colonies on this medium, whereas Gram staining confirmed that the isolate was Gram-negative (Figure-1c). Pure isolates presumed to be *K. pneumoniae* were further confirmed by subculturing onto EMBA followed by incubation at 37°C for 24 h. *K. pneumoniae* colonies appeared pinkish-black with a mucoid texture on EMBA (Figure-1b). The confirmed isolates were subsequently subjected to biochemical identification using several differential media. On TSIA, *K. pneumoniae* showed a yellow (acidic) color change with gas production but was negative for hydrogen sulfide (H_2_S) production. The urease test showed a positive reaction, with the medium turning pink, indicating urease enzyme activity. In SIM medium, the isolates were negative for H_2_S and indole production and were non-motile. The Methyl Red test was negative, whereas the Voges–Proskauer (VP) test was positive, indicating acetoin production. On Simmons citrate agar (SCA), a positive reaction, indicated by a blue coloration, demonstrated the ability to utilize sodium citrate as a carbon source and ammonium phosphate as a nitrogen source. The biochemical test results confirming the presence of *K. pneumoniae* are presented in Figure 2.

### Antimicrobial susceptibility test results

Pure *K. pneumoniae* isolates were tested for antimicrobial susceptibility using the Kirby–Bauer disk diffusion method on Mueller–Hinton Agar (MHA) (Oxoid). The selected antibiotics represent several antimicrobial classes relevant to dairy cattle treatment, environmental exposure, and public health, supporting a strong MDR classification in line with the One Health surveillance goals. Seven antibiotics commonly used in veterinary and dairy practices in Indonesia were tested, representing different antimicrobial classes, including ampicillin (AMP; 10 μg), cefotaxime (CTX; 30 μg), tetracycline (TE; 30 μg), ciprofloxacin (CIP; 5 μg), streptomycin (S; 10 μg), trimethoprim–sulfamethoxazole (STX; 1.15/23.75 μg), and chloramphenicol (C; 30 μg), all obtained from Oxoid, UK [28, 34].

**Figure 3:**
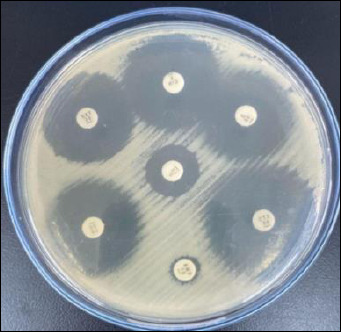
Confirmatory test of antibiotic sensitivity by disk diffusion with the Kirby–Bauer method on Mueller–Hinton agar media.

Antibiotic disks were placed on the agar surface with a center-to-center distance of approximately 25–30 mm, followed by incubation at 35°C ± 2°C for 16–18 h (Figure 3) [35]. Antimicrobial susceptibility testing was performed according to the 2020 Clinical and Laboratory Standards Institute (CLSI) guidelines, including standardized inoculum preparation adjusted to the 0.5 McFarland turbidity standard and controlled incubation conditions.

Although a reference quality-control strain (e.g., *Escherichia coli* ATCC 25922) was not included during routine testing, strict adherence to CLSI-recommended procedures was applied to minimize technical variability. Isolates resistant to ≥3 antimicrobial classes were classified as MDR [36].

### ESBL confirmation test

**Figure 4:**
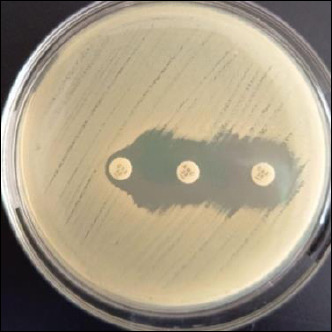
Positive results of extended-spectrum β-lactamase-producing bacteria on the double-disk synergy test.

The double-disk synergy test (DDST) is a phenotypic assay used to identify *K. pneumoniae* isolates that produce ESBL [37]. MDR isolates were tested with the Kirby–Bauer disk diffusion method on MHA following CLSI guidelines [28]. This test involved ceftazidime (CAZ; 30 μg), cefotaxime (CTX; 30 μg), and amoxicillin–clavulanate (AMC; 30/10 μg), placed 20 mm apart from the amoxicillin–clavulanate disk [35]. Positive ESBL results were confirmed by the formation of a clear zone with a characteristic keyhole pattern between β-lactam antibiotic disks and AMC [28]. ESBL confirmation was performed using *E. coli* ATCC 25922 as a negative control and *K. pneumoniae* ATCC 700603 as a positive control (Figure 4).

### Quality assurance

Quality assurance procedures were followed during sample collection, transportation, and laboratory analysis to ensure data accuracy and consistency in methods. All sampling equipment and laboratory consumables were sterilized and used only once per sample to prevent cross-contamination. Culture media and reagents were prepared according to the manufacturer’s instructions and ISO and FDA BAM guidelines. Media sterility was confirmed by incubating uninoculated control plates alongside test samples.

Laboratory instruments, including incubators and refrigerators, were routinely calibrated and monitored to ensure proper functioning. Bacterial suspensions used for antimicrobial susceptibility testing were standardized with a 0.5 McFarland turbidity standard. Quality control for antimicrobial susceptibility testing was conducted according to CLSI guidelines. A randomly selected subset of isolates was re-tested to confirm reproducibility. All laboratory procedures were performed by trained personnel following standardized operating procedures. Data entry and laboratory records were cross-checked to reduce transcription errors and ensure consistency between laboratory results and analytical datasets.

### Statistical analysis

Data were analyzed descriptively. Prevalence estimates were calculated as proportions and presented with 95% confidence intervals. Binomial proportion confidence intervals were calculated to quantify the precision of the prevalence estimates. Statistical analyses were performed using Microsoft Excel version 21 (Microsoft Corp., Redmond, WA, USA).

## RESULTS

### Isolation and phenotypic identification of *K. pneumoniae* strains

Isolation and phenotypic identification of samples collected from 59 dairy farms in Batu City showed that *K. pneumoniae* was found in 61.3% of all samples (217/354; 95% CI: 56.1–66.3). The prevalence of *K. pneumoniae* differed across sample types. The highest prevalence was seen in forage feed (84.7%; 50/59; 95% CI: 73.5–92.4), followed by animal drinking water (74.6%; 44/59; 95% CI: 62.9–83.8), hand-wash water (71.2%; 42/59; 95% CI: 59.1–81.2), and soil (71.2%; 42/59; 95% CI: 59.1–81.2). A moderate prevalence was found in milk samples (50.8%; 30/59; 95% CI: 37.6–64.0), while the lowest prevalence appeared in fecal samples (15.3%; 9/59; 95% CI: 7.3–27.0) (Table 1).

**Table 1:** Identification of *Klebsiella pneumoniae*.

Sample type	Total samples (n)	Positive n (%)	95% Confidence interval
Hand-wash water	59	42 (71.2)	59.1–81.2
Animal drinking water	59	44 (74.6)	62.9–83.8
Milk	59	30 (50.8)	37.6–64.0
Soil	59	42 (71.2)	59.1–81.2
Forage feed	59	50 (84.7)	73.5–92.4
Feces	59	9 (15.3)	7.3–27.0
Total	354	217 (61.3)	56.1–66.3

### Antibiotic susceptibility test results

Antimicrobial susceptibility testing of *K. pneumoniae* isolates showed varying resistance patterns to different antimicrobial agents (Table 2). The highest resistance was seen with ampicillin (89.4%; 194/217), followed by streptomycin (71.4%; 155/217) and cefotaxime (38.3%; 83/217). Lower resistance rates were noted for sulfamethoxazole–trimethoprim (12.4%; 27/217), chloramphenicol (12.4%; 27/217), tetracycline (9.2%; 20/217), and ciprofloxacin (2.8%; 6/217). Resistance profiles varied among different sample types. Isolates from forage feed generally showed higher resistance rates to several antibiotics, especially ampicillin, streptomycin, and cefotaxime, compared to isolates from other sources. In contrast, resistance to ciprofloxacin and tetracycline remained consistently low across all sample types (Table 2).

**Table 2:** Antimicrobial resistance patterns of *Klebsiella pneumoniae* isolates by sample type.

AMR data	Hand-wash water (n = 42)	Animal drinking water (n = 44)	Milk (n = 30)	Soil (n = 42)	Forage feed (n = 50)	Feces (n = 9)	Subtotal (n = 217)
**CTX**							
R	17 (40.5)	15 (34.1)	12 (40)	14 (33.3)	23 (46)	2 (22.2)	83 (38.3)
I	7 (16.7)	9 (20.4)	4 (13.3)	11 (26.2)	11 (22)	1 (11.1)	43 (19.8)
S	18 (42.8)	20 (45.5)	14 (46.67)	17 (40.5)	16 (32)	6 (66.7)	91 (41.9)
C							
R	3 (7.1)	3 (6.8)	7 (23.33)	6 (14.3)	8 (16)	0 (0)	27 (12.5)
I	7 (16.7)	5 (11.4)	1 (3.33)	3 (7.1)	5 (10)	2 (22.2)	23 (10.6)
S	32 (76.2)	36 (81.8)	22 (73.33)	33 (78.6)	37 (74)	7 (77.8)	167 (76.9)
**AMP**							
R	37 (88.1)	42 (95.5)	25 (83.33)	37 (88.1)	45 (90)	8 (88.9)	194 (89.4)
I	5 (11.9)	2 (4.5)	3 (10)	1 (2.4)	3 (6)	1 (11.1)	15 (6.9)
S	0 (0)	0 (0)	2 (6.66)	4 (9.5)	2 (4)	0 (0)	8 (3.7)
**TE**							
R	3 (7.1)	5 (11.4)	5 (16.7)	3 (7.1)	4 (8)	0 (0)	20 (9.2)
I	1 (2.4)	1 (2.2)	0 (0)	0 (0)	1 (2)	0 (0)	3 (1.4)
S	38 (90.5)	38 (83.4)	25 (83.33)	39 (92.9)	45 (90)	9 (100)	194 (84.4)
S							
R	25 (59.5)	31 (70.5)	22 (73.33)	31 (73.8)	38 (76)	8 (88.9)	155 (71.4)
I	11 (26.2)	11 (25)	6 (20)	7 (16.7)	9 (18)	1 (11.1)	45 (20.8)
S	6 (14.3)	2 (4.5)	2 (6.66)	4 (9.5)	3 (6)	0 (0)	17 (7.8)
**SXT**							
R	6 (14.3)	5 (11.4)	5 (16.7)	6 (14.3)	4 (8)	1 (11.1)	27 (12.5)
I	2 (4.8)	2 (4.5)	1 (3.33)	5 (11.9)	0 (0)	0 (0)	10 (4.6)
S	34 (80.9)	37 (84.1)	24 (80)	31 (73.8)	46 (92)	8 (88.9)	180 (82.9)
**CIP**							
R	1 (2.4)	0 (0)	0 (0)	2 (4.8)	3 (6)	0 (0)	6 (2.8)
I	1 (2.4)	2 (4.5)	1 (3.33)	0 (0)	1 (2)	0 (0)	5 (2.3)
S	40 (95.2)	42 (95.5)	29 (96.67)	40 (95.2)	46 (92)	9 (100)	206 (94.9)

AMP = Ampicillin, AMR = Antimicrobial resistance, C = Chloramphenicol, CIP = Ciprofloxacin, CTX = Cefotaxime, I = Intermediate, R = Resistant, S = Streptomycin, S = Susceptible, SXT = Sulfamethoxazole–trimethoprim, TE = Tetracycline.

Isolates resistant to three or more classes of antimicrobial agents were classified as MDR [38]. Among the 217 *K. pneumoniae* isolates, 35.0% (76/217) were MDR (Table 3). Resistance to three antimicrobial classes was the most common MDR pattern, accounting for 61.8% (47/76) of MDR isolates, followed by resistance to five classes (21.1%; 16/76), four classes (14.5%; 11/76), and six classes (2.6%; 2/76). No isolates showed resistance to any of the seven antimicrobial classes tested. The distribution of MDR isolates across sample types varied based on the number of resistant antimicrobial classes. MDR isolates were most often found in forage feed, hand-wash water, and animal drinking water, while isolates resistant to five or six antimicrobial classes were mainly detected in soil and milk samples (Table 3).

**Table 3:** Distribution of MDR *Klebsiella pneumoniae* isolates by number of resistant antimicrobial classes and sample type (n = 76).

Antimicrobial classes	Hand-wash water	Animal drinking water	Milk	Soil	Forage feed	Feces	Subtotal n (%)
3	13 (27.7)	10 (21.3)	5 (10.5)	6 (12.8)	11 (23.4)	2 (4.3)	47 (61.8)
4	0 (0.0)	4 (36.4)	2 (18.2)	0 (0.0)	5 (45.4)	0 (0.0)	11 (14.5)
5	2 (12.5)	1 (6.3)	2 (12.5)	7 (43.7)	4 (25.0)	0 (0.0)	16 (21.1)
6	0 (0.0)	0 (0.0)	2 (100)	0 (0.0)	0 (0.0)	0 (0.0)	2 (2.6)
7	0 (0.0)	0 (0.0)	0 (0.0)	0 (0.0)	0 (0.0)	0 (0.0)	0 (0.0)
Total							76 (100)

MDR = Multidrug-resistant.

MDR was defined as resistance to three or more classes of antimicrobial agents. Percentages within rows represent the distribution of MDR isolates across sample types for each resistance category. Subtotal percentages represent the overall distribution of MDR isolates. Percentages were calculated using the total number of MDR isolates (n = 76) as the denominator. Values 3–7 indicate the number of different antimicrobial classes to which isolates exhibit resistance.

### ESBL confirmation test

Among the 76 MDR *K. pneumoniae* isolates, 21.1% (16/76; 95% CI: 13.1–31.9) were confirmed as ESBL producers using DDST (Table 4). The highest prevalence of ESBL-producing isolates was found in forage feed samples (7.9%; 6/76; 95% CI: 3.7–15.9), followed by animal drinking water (6.6%; 5/76; 95% CI: 2.9–14.5), soil (3.9%; 3/76; 95% CI: 1.3–10.8), hand-wash water (1.3%; 1/76; 95% CI: 0.2–7.1), and milk (1.3%; 1/76; 95% CI: 0.2–7.1). No ESBL-producing isolates were detected in fecal samples (0%; 95% CI: 0.0–4.8).

**Table 4:** Prevalence of ESBL-producing *Klebsiella pneumoniae* among MDR isolates confirmed by DDST.

Sample type	ESBL-positive (n)	Prevalence (%)	95% CI
Hand-wash water	1	1.3	0.2–7.1
Animal drinking water	5	6.6	2.9–14.5
Milk	1	1.3	0.2–7.1
Soil	3	3.9	1.3–10.8
Forage feed	6	7.9	3.7–15.9
Feces	0	0	0.0–4.8
Total	16	21.1	13.1–31.9

Prevalence was calculated based on the total number of MDR *K. pneumoniae* isolates (n = 76). CI = Confidence interval, DDST = Double-disk synergy test, ESBL = Extended-spectrum β-lactamase, MDR = Multidrug-resistant.

## DISCUSSION

### Occurrence of *K. pneumoniae* in dairy farm matrixes

This study shows a high presence of *K. pneumoniae* across various matrixes within dairy cattle farms in Batu City, suggesting widespread environmental contamination throughout the farm ecosystem. *K. pneumoniae* was found in more than half of all samples collected, emphasizing the extensive circulation of this organism across animal-related, environmental, and human-contact interfaces. The dominance of *K. pneumoniae* in forage feed, drinking water, hand-wash water, and soil highlights the significance of non-animal matrixes as key reservoirs that may support bacterial survival and spread within dairy production systems [39–41].

### Comparison with previous studies and possible sources of contamination

Compared with previous studies conducted in the Republic of the Congo and China, which reported much lower *K. pneumoniae* detection rates, the higher occurrence observed in this study suggests differences in context related to farm management practices, environmental hygiene, and patterns of antimicrobial use [6, 42]. While variations in sampling design and matrixes affect direct comparisons across studies, the consistently high detection across different sample types in this study indicates that dairy farms in Batu City represent a complex microbial ecosystem where *K. pneumoniae* is widespread. This finding is especially relevant in tourism-integrated dairy farms, where frequent interactions between animals, farm environments, and the public may heighten exposure and cross-contamination risks [43].

### Antimicrobial resistance patterns in dairy farm isolates

Antimicrobial susceptibility testing showed high resistance levels to antibiotics commonly used in food-producing animals, especially ampicillin and streptomycin. These results agree with reports from Indonesia and other Southeast Asian nations, where penicillins, sulfonamides, tetracyclines, and quinolones are frequently used in livestock production [24]. Studies from Southeast Asia consistently indicate that extensive and sometimes inappropriate use of antimicrobials in dairy farming leads to higher resistance rates, particularly to β-lactam antibiotics. The similarity between the resistance patterns observed in this study and those reported in wastewater surveillance suggests that dairy farms serve as significant upstream sources of AMR, contributing to environmental contamination downstream [28]. However, unlike wastewater research, the current farm-level, multimatrix approach allows for earlier detection of resistance sources before they are diluted and dispersed.

### Multidrug resistance and antimicrobial usage pressure

The proportion of MDR *K. pneumoniae* identified in this study further highlights the severity of AMR in dairy farm environments. Differences in MDR prevalence reported across various regions may reflect variations in sample matrixes, animal health, herd management, and antimicrobial use [44–46]. In East Java, where dairy cattle populations and antibiotic use are among the highest in Indonesia, selective pressure from frequent antimicrobial application may significantly contribute to MDR emergence [47]. The use of antibiotics for prophylaxis, metaphylaxis, and nonbacterial conditions, including agents classified as veterinary critically important antimicrobial agents, likely worsens the selection and persistence of resistant strains [24, 47].

### Distribution of ESBL-producing isolates in environmental matrixes

The detection of ESBL-producing *K. pneumoniae* among MDR isolates raises an additional public health concern. ESBL producers were mainly found in environmental samples, especially forage feed and water sources, rather than being limited to animal-derived specimens. This distribution highlights the farm environment as a key point for the spread of AMR. Feed contamination, in particular, can occur through contact with soil, water, or poorly sanitized storage facilities, leading to repeated exposure of animals to resistant bacteria [48, 49]. These findings indicate that feed management and storage practices are critical points for controlling AMR spread within dairy systems.

### Environmental persistence of ESBL-producing bacteria

The environmental persistence of ESBL-producing *K. pneumoniae* may further contribute to its widespread spread. *K. pneumoniae* is known for its ability to survive in various environmental conditions, including soil and water, and to form biofilms that boost resistance to environmental stressors and disinfectants [50, 51]. This persistence allows resistant strains to stay viable in farm environments even without direct antimicrobial pressure, increasing the chance of long-term survival and circulation. The presence of ESBL-producing isolates in multiple environmental compartments suggests that dairy farms could act as stable reservoirs supporting the persistence of antimicrobial resistance [52].

### Horizontal gene transfer and transmission risk within-farm ecosystems

Beyond persistence, the co-occurrence of MDR and ESBL-producing *K. pneumoniae* in environmental matrixes raises concerns about horizontal gene transfer (HGT). ESBL genes are often found on mobile genetic elements, such as plasmids, which can be exchanged through conjugation, transformation, or transduction among bacterial populations [19, 53]. Environments with high microbial density, like contaminated feed, water, and soil, offer favorable conditions for genetic exchange [54]. Therefore, the detection of ESBL producers across environmental, animal-associated, and human-contact matrixes indicates an increased potential for interspecies and intercompartmental spread of resistance determinants within dairy farm ecosystems.

### One Health implications of MDR and ESBL dissemination

The concurrent detection of MDR- and ESBL-producing *K. pneumoniae* across animal-, environmental-, and human-related interfaces underscores the interconnected transmission pathways within dairy farm ecosystems. These results emphasize the need to adopt a One Health approach, recognizing that AMR control extends beyond animals to include feed safety, water quality, environmental sanitation, and responsible antimicrobial use [43, 55]. The risk of zoonotic and environmental transmission in tourism-linked dairy farms, where livestock production overlaps with public access, further heightens the public health significance of these findings.

### Novel contributions and significance of the present study

Overall, this study offers several new contributions to research on AMR in dairy production systems. It provides a farm-level, multimatrix assessment of *K. pneumoniae* that identifies upstream resistance reservoirs often missed in clinical or wastewater studies. Finding forage feed as a key reservoir of ESBL-producing *K. pneumoniae* highlights an overlooked pathway for spreading resistance. Additionally, by examining animal-derived, environmental, and human-related matrixes simultaneously, the study reveals interconnected transmission interfaces aligned with a One Health approach. Although ESBL detection was limited to phenotypic methods, the results give valuable baseline data for Indonesian dairy farms and underline the need for future molecular and longitudinal research to better understand resistance patterns and guide targeted intervention efforts [52, 56, 57].

### Limitations and future research directions

This study has several limitations to consider when interpreting the results. First, the cross-sectional design offers a snapshot of AMR at a single point in time and cannot assess changes over time or seasonal variations in the occurrence and resistance patterns of *K. pneumoniae*. Second, ESBL detection depended solely on phenotypic confirmation with DDST, without molecular analysis of ESBL-related genes. The lack of molecular typing restricts the ability to identify specific resistance genes, clonal relationships, and transmission pathways.

Third, the study was conducted in a single geographic area and involved one-time sampling per farm, which may limit the applicability of the findings to other dairy production systems with different management practices or environmental conditions. Additionally, although environmental and animal-associated matrixes were thoroughly assessed, human carriage was not evaluated, preventing direct conclusions about zoonotic transmission to farm workers or visitors. Furthermore, the study did not quantitatively analyze farm-level risk factors, such as hygiene practices, water source quality, or feed storage conditions, which may affect the distribution and persistence of AMR bacteria.

This study relied on phenotypic AMR testing following the CLSI guidelines; however, the lack of a reference quality-control strain in the AMR test (e.g., *E. coli* ATCC 25922) is a methodological limitation that should be addressed in future studies to further enhance laboratory quality assurance.

Despite these limitations, the current study provides valuable baseline data on the prevalence of MDR and ESBL-producing *K. pneumoniae* across different matrixes within dairy farm environments. Future research should focus on longitudinal and seasonal sampling to identify temporal trends, incorporate molecular techniques to analyze resistance genes and mobile genetic elements, and include human sampling to better understand zoonotic and environmental transmission pathways. Combining farm-level risk factor assessments with whole-genome sequencing would further enhance One Health–based surveillance and support targeted intervention strategies to reduce AMR in dairy production systems.

## CONCLUSION

This study showed a high presence of *K. pneumoniae* across various matrixes in dairy farm environments in Batu City, with detection in more than half of all samples collected. The highest prevalence was found in forage feed, drinking water, hand-wash water, and soil. Antimicrobial susceptibility testing indicated high resistance rates to commonly used antibiotics, especially ampicillin, streptomycin, and cefotaxime. Over one-third of the isolates were classified as MDR. Among these MDR isolates, a significant portion were confirmed as ESBL producers, mainly found in environmental matrixes rather than solely in animal-derived samples. These results suggest that dairy farms are complex ecosystems where animal, environmental, and human interactions help sustain and spread antimicrobial resistance.

From a practical perspective, the dominance of MDR and ESBL-producing *K. pneumoniae* in feed, water, and soil emphasizes the need to improve farm hygiene, feed storage practices, water quality monitoring, and responsible antimicrobial use to lower the risk of resistance spreading within dairy production systems. The detection of environmental matrixes as key reservoirs indicates that focusing solely on animal treatment is not enough, and comprehensive control measures must include environmental sanitation and antimicrobial stewardship. These findings are especially important for dairy farms that also serve as tourist destinations, where frequent contact between livestock, farm environments, and visitors may raise the risk of cross-contamination and public exposure.

A key strength of this study is its multimatrix, farm-level surveillance method, which enabled the detection of upstream reservoirs of MDR and ESBL-producing bacteria that are often missed in clinical or wastewater studies. By analyzing milk, feces, feed, soil, drinking water, and hand-wash water simultaneously, this research offers a more comprehensive understanding of resistance spread within dairy farm environments and supports using a One Health approach for AMR surveillance.

In conclusion, dairy farm environments in Batu City may act as key reservoirs for MDR and ESBL-producing *K. pneumoniae*, with possible effects on animal health, environmental contamination, and public health. Enhancing antimicrobial stewardship, improving environmental hygiene, and implementing integrated One Health surveillance programs are crucial to limit the emergence and spread of AMR in dairy production systems. Further longitudinal and molecular studies are necessary to better understand transmission pathways and to develop targeted strategies for sustainable AMR management.

## DATA AVAILABILITY

The datasets generated and/or analyzed during the current study are available from the corresponding author upon reasonable request.

## AUTHORS’ CONTRIBUTIONS

FNAEPD: Conceptualization, study design, supervision of laboratory work, critical review of the manuscript, and corresponding author responsibility. SMY: Study design, data interpretation, and manuscript revision. ENU: Antimicrobial resistance interpretation and critical revision of the manuscript. ARK: Antimicrobial resistance interpretation and manuscript revision. MHE: Study design, data interpretation, and critical revision of the manuscript. RS: Data analysis, data interpretation, drafting of the manuscript, and manuscript revision. MAAS: Sample collection and preliminary data processing. IAA, SK, and SLA: Supervision of laboratory work and critical review of the manuscript. TE, NYN, BAM, and NAPD: Field sampling, data entry, laboratory analyses, bacterial isolation, bacterial identification, and antimicrobial susceptibility testing. All authors have read and approved the final version of the manuscript.
